# Protease enzyme in broiler diets on the nutritional quality of soybean meals from different origins

**DOI:** 10.1007/s11250-026-04851-y

**Published:** 2026-01-16

**Authors:** Diego Ladeira da Silva, Felipe Santos Dalólio, Jean Kaique Valentim, Rosa Aparecida Reis de Léo, Helder Freitas de Oliveira, Levy do Vale Teixeira, Túlio Leite Reis, Vitor Barbosa Fascina, Arele Arlindo Calderano

**Affiliations:** 1 Department of Animal Science, Federal University of Viçosa (UFV), Minas Gerais Viçosa, 36.570-900 Brazil; 2 Department of Animal Production, Federal University of Rural Rio de Janeiro (UFRRJ), Seropédica, Rio de Janeiro 23.897-000 Brazil; 3 Department of Animal Science, Federal University of Goiás (UFG), Goiânia, Goiás 74.690-900 Brazil; 4 Innovation and Applied Science, DSM Nutritional Products Brazil, Mairinque, São Paulo 18.120-000 Brazil

**Keywords:** Metabolizable energy, Digestible amino acids, Ileal digestibility, Soybean meal

## Abstract

The objective of this study was to evaluate the apparent metabolizable energy (AME), nitrogen-corrected apparent metabolizable energy (AMEn), digestible amino acids, and true ileal digestibility coefficient (TIDC) of soybean meals (SMs) from different origins, with and without the addition of protease to broiler diets. The experiment using 756 male Cobb broiler chicks, in a completely randomized design with a 10 × 2 + 1 factorial arrangement (reference diet), totaling 21 treatments with 6 replications of 6 birds each. To determine the values of AME and AMEn, the diets were provided *ad libitum* for 10 days, with total excreta collected during the last 5 days. The protease was added on top. For the analysis of digestible amino acids and TIDC, a basal protein-free diet was used to determine the endogenous losses of amino acids in the ileum of the birds. The results revealed significant variations in the AME and AMEn values among the soybean meals, with the addition of protease promoting increases in the AME and AMEn values. Additionally, significant differences were observed in the digestible amino acid values, which were influenced by the origin and processing of the soybean meals. Protease supplementation also improved amino acid digestibility, especially for the main essential and nonessential amino acids. In conclusion, soybean meals from different regions exhibit significant variations in terms of metabolizable energy and digestible amino acids, and the addition of protease improves both the digestibility and true ileal digestibility coefficients, resulting in increased AME and AMEn values.

## Introduction

The assessment of the nutritional quality of ingredients used in broiler diets is crucial for optimizing animal performance and reducing production costs, particularly in tropical and subtropical regions. Soybean meal stands out as one of the main protein sources in poultry diets because of its balanced amino acid profile and high metabolizable energy content, making it essential for ensuring proper nutrition for poultry (Silva et al. 2022; Pires et al. 2022; Xavier et al. 2024).

However, the nutritional composition and digestibility of nutrients in soybean meal can vary considerably depending on geographic origin and industrial processing, which can directly impact feed efficiency and animal performance. These variations make the evaluation and optimization of the nutritional value of soybean meal a critical factor for success in poultry feeding (Ravindran et al. 2014; Silva et al. 2022).

In addition to natural variations among soybean meals, the presence of antinutritional factors, such as trypsin inhibitors, lectins, and phytates, can reduce the bioavailability of nutrients, impairing the digestion and absorption of amino acids and energy. These antinutritional factors limit the efficient utilization of feed ingredients, which may compromise the performance of poultry (Carvalho et al. 2020; Khajali and Rafiei 2024). The toasting temperature of the bran can also alter product quality, with high temperatures reducing protein digestibility, while lower temperatures may be sufficient to inactivate antinutritional factors. Therefore, some analyses are typically performed by the industry to verify the quality of soybean meal (Dozier and Hess 2011).

To overcome these limitations, the use of exogenous enzymes, such as proteases, has proven to be an effective strategy to improve nutrient digestibility and increase feed efficiency in broilers (Santos Neto et al. 2021). Therefore, enzyme supplementation has emerged as a promising tool for optimizing diets, particularly in conditions where ingredient quality is variable (Adeola and Cowieson 2011; Dalólio et al. 2016a; Cho et al. 2020).

One particularly promising enzyme in this context is the protease, which is derived from the fermentation of *Bacillus licheniformis*. Studies indicate that this protease can increase the availability of amino acids and energy in poultry diets, contributing to improved feed efficiency (Cowieson and Roos 2016; Xavier et al. 2024; Liu et al. 2024). The literature shows that protease supplementation can lead to significant improvements in nutrient digestibility and animal performance, especially by facilitating the breakdown of complex proteins into simpler and more absorbable forms. This, in turn, enhances the efficiency of protein utilization in the diet, a crucial factor for poultry performance (Ghazi et al. 2002; Odetallah et al. 2002; Dalólio et al. 2016b).

Furthermore, improvements in apparent metabolizable energy (AME) and nitrogen-corrected apparent metabolizable energy (AMEn) levels have been consistently observed in diets supplemented with proteases, reinforcing the effectiveness of this strategy (Bernardes et al. 2022; Carneiro et al. 2023).

In this context, the present study was designed with the aim of evaluating the values of AME, AMEn, digestible amino acids, and true ileal digestibility coefficients (TIDCs) of soybean meals from different origins, with and without the addition of the protease protease.

## Materials and methods

All procedures adopted in this study were previously evaluated and approved by the Ethics Committee on the Use of Farm Animals (Registration Protocol: 47/2017) and followed the ethical principles of animal experimentation established by the National Council for Animal Experimentation Control (CONCEA).

A total of 756 male Cobb-500^®^ broilers were raised until 14 days of age with an average weight of 470 g in a masonry shed divided into protected circular pens. These pens contained sawdust bedding, tubular feeders, and manual drinkers, with free access to feed and water. The lighting program consisted of 24 h of light, and the temperature was maintained at 32 °C during the first week and was gradually reduced according to the Cobb^®^ manual recommendations. The diets, which was based on corn and soybean meal, was formulated to meet the nutritional recommendations proposed by Rostagno et al. (2017).

At 14 days of age, the birds were weighed and distributed into a completely randomized design to determine the apparent metabolizable energy (AME), nitrogen-corrected apparent metabolizable energy (AMEn), and apparent and standardized digestibility coefficients of amino acids.

The birds were transferred to wire floor cages (500 cm²/bird) in a four-level battery equipped with a trough-type feeder and nipple-type drinker. The experimental period lasted from the 14th to the 28th day of life. The treatments were formulated by adding nine different soybean meals to the basal diet.

The dry matter (DM), gross energy (GE), and crude protein (CP) contents of soybean meals from different regions of Brazil were evaluated. Soybean meal samples were collected from ten different feed mills in Brazil. The samples were collected directly from producers in the respective regions, packed in sealed plastic bags. The samples were then transported to the laboratory under controlled conditions to avoid contamination and degradation, and the results are expressed as percentages (Table 1).

**Table 1:** Analyzed values of dry matter (DM), gross energy (GE) and crude protein (CP) of soybean meal from different regions

Soybean meal	DM, %	GE, Kcal/kg de MN	CP, %
1	88.23	4127	45.10
2	88.34	4144	43.88
3	88.35	3960	46.55
4	88.37	4116	47.79
5	88.07	4142	45.40
6	88.21	4129	46.02
7	88.10	4093	44.46
8	88.43	4165	45.01
9	88.52	4139	42.43
10	88.36	4090	46.70

The enzyme protease commercial RONOZYME^®^ ProAct was used on top. The protease supplementation was performed using an exogenous protease at the manufacturer’s recommended dose at 15,000 protease per kg of feed. One protease unit is defined as the amount of enzyme that releases 1 µmol of p-nitroaniline (pNA) from a 1 mmol/L (N-Suc-Ala-Ala-Pro-Phe pNA) substrate per minute at pH 9.0 and 37 °C. This protease is produced through the fermentation of *Bacillus licheniformis*, which contains transcribed genes from *Nocardiopsis prasina*. The enzymatic activity of this enzyme is defined by the amount of enzyme required to degrade 1 µmol of p-nitroaniline from 1 µM of the substrate (Suc-Ala-Ala-Pro-Phe-N-succinyl Ala-Ala-Pro-Phe-p-nitroanilide) per minute at a pH of 9.0 and 37 °C. The product used contains 75,000 protease units/g of enzyme.

The soybean meal samples were analyzed for key nutritional and antinutritional parameters, including crude protein (CP), soluble protein percentage, KOH solubility, trypsin inhibitor activity (TIA), urease activity (UA), and trypsin inhibitors concentration (IT). The crude protein content was measured using the Kjeldahl method (AOAC, 1995), where total nitrogen content is multiplied by a factor of 6.25 to estimate crude protein as a percentage of dry matter. Soluble protein and KOH solubility were determined according to the method described by Araba and Dale (1990), which involves measuring the protein fraction that dissolves in a 0.2% potassium hydroxide solution.

The trypsin inhibitor activity (TIA) was quantified based on the method outlined by Hamerstrand et al. (1981), with results expressed in milligrams of trypsin inhibitors per gram of soybean meal (mg TI/g). Urease activity (UA) was assessed by measuring the pH variation (Δ pH) after mixing the sample with a urea solution, following the Ba 9–58 method from the American Oil Chemists’ Society (AOCS, 2017).

The concentration of trypsin inhibitors (IT) was measured as part of the TIA analysis and expressed in milligrams per gram (mg IT/g). The protein dispersibility index (PDI) was determined according to the Ba 10–65 method from AOCS (2017) using a Hamilton blender (Model G936, VOS Instrument, Zaltbommel, Netherlands). These parameters were analyzed in triplicate for each sample to ensure reliability and consistency of the results.

### Experiment 1. Metabolizable energy values of soybean from different origins, with and without the addition of protease

The diets were provided *ad libitum* for a 10-day period (from 14 to 23 days of age), with the first five days allocated for adaptation and the last five days dedicated to the total fecal collection methodology. Fecal collection was conducted twice a day at twelve-hour intervals to prevent fermentation and nutrient loss.

The birds were given a basal diet formulated with corn and soybean meal containing 20.8% crude protein (CP) and 3000 kcal of metabolizable energy (ME)/kg of feed (Table 2). The experimental diets consisted of 70% of the basal diet and 30% of the soybean meal to be evaluated for each treatment.

**Table 2:** Percentage composition of the reference ration

Ingredients	%
Corn	59.070
Soybean meal	34.760
Soybean oil	2.170
Bicálcyc phosphate	1.527
Limestone	0.912
Common salt	0.482
L-lisina HCl (98%)	0.213
DL-metionina (99%)	0.285
Vitamin Supplement ^1^	0.110
Mineral Supplement ^2^	0.110
Choline chloreth (60%)	0.100
Salinomycin (12%) ^3^	0.050
Avilamicina (10%) ^4^	0.010
Antioxidant ^5^	0.010
**Total**	**100.00**
**Calculated composition**	
Metabolizable energy (kcal/kg)	3.000
Gross protein (%)	20.80
Digestible lysine (%)	1.174
Digestible methionine (%)	0.562
Digestible methionine + cystine (%)	0.846
Digestible threonine (%)	0.763
Digestible tryptophan (%)	0.231
Calcium (%)	0.819
Available Phosphorus (%)	0.394
Sodium (%)	0.210

1Composition per kg of feed: vit. A, 8.250 IU; vit. D3, 2,090 IU; vit. AND 31 IU; vit. B1, 2.20 mg; vit B2, 5.50 mg; vit. B6, 3.08 mg; ac. pantothenic, 11.0 mg; biotin, 0.077 mg; vit. K3, 1.65 mg; folic acid, 0.770 mg; nicotinic acid, 33.0 mg; vit. B12, 0.013 mg; selenium, 0.330 mg. 2 Composition per kg of feed: manganese, 77.0 mg; iron, 55.0 mg; zinc, 71.5 mg; copper, 11.0 mg; iodine, 1.10 mg.3 Anticoccidial (Coxistac)0.4 Growth promoter (Surmax)0.5 Butyl Hydroxy Toluene (BHT)

The excreta collected from each experimental unit were placed in appropriately labeled plastic bags and stored in a freezer (-18 °C) until the end of the collection period (5 days). At the end of the experimental period, the collected feces were thawed, weighed, homogenized, and dried in a ventilated oven at 55 °C for 72 h.

The values of dry matter (DM), nitrogen (N), and gross energy (GE) were determined in the soybean meal samples, experimental diets, and feces. Dry matter was obtained by oven drying, nitrogen content was measured using the Kjeldahl method, and gross energy was quantified using a bomb calorimeter, following standard procedures described by Sakomura and Rostagno (2016). Based on these analytical values, the apparent metabolizable energy (AME) of the soybean meal samples was calculated using the classical difference between ingested and excreted energy. The nitrogen-corrected apparent metabolizable energy (AMEn) was obtained by adjusting AME for nitrogen retention, applying the correction factor of 8.22 kcal per gram of retained nitrogen, as recommended by Sakomura and Rostagno (2016).

### **Experiment 2: Digestible amino acids and true ileal digestibility coefficients of soybean meals from different origins**,** with and without the addition of protease**

In this study, 756 24-day-old male Cobb 500 broilers, with an average weight of 880 ± 10 g, were used. At 23 days of age, the birds were transferred to metal battery cages and distributed in a completely randomized design with a 10 × 2 ± 1 factorial arrangement (a protein-free basal diet (DIP) based on starch, with the inclusion of 30% of each of the 10 soybean meals from different regions, with and without the addition of protease), totaling 21 treatments with 6 repetitions of 6 birds per cage. The birds were housed from the 24th to the 28th day of age.

A protein-free diet (DIP) was formulated to determine the endogenous losses of amino acids in the ileum of the birds (Table 3). Insoluble ash (Celite™) was added at a concentration of 1% to all experimental diets to quantify the endogenous losses and calculate the digestibility coefficients.

**Table 3:** Composition of experimental diets

Ingredients	Protein-free diet (%)	Protein-free diet + 30% soybean meal (%)
Starch	80.310	50.310
Soybean meal (1 to 10)	-	30.000
Sugar	5.000	5.000
Soybean oil	5.000	5.000
Dicalcium phosphate	2.100	2.100
Limestone	0.700	0.700
Salt	0.450	0.450
Potassium carbonate	1.000	1.000
Corn cob	4.000	4.000
Vitamin supplement ^1^	0.080	0.080
Mineral supplement ^2^	0.150	0.150
Choline chloreth (60%)	0.200	0.200
Antioxidant3	0.010	0.010
Insoluble acid ash (Celite^TM)^	1.000	1.000
Total	100.000	100.000

1Composition per kg of product: vit. A, 12,000,000 IU; vit. D3, 2,200,000 IU; vit. AND 30,000 IU; vit. B1, 2,200 mg; vit B2, 6,000 mg; vit. B6, 3,300 mg; ac. pantothenic, 13,000 mg; biotin, 110 mg; vit. K3, 2,500 mg; folic acid, 1,000 mg; nicotinic acid 53.0000 mg; niacin, 25,000 mg; vit. B12, 16,000 μg; selenium, 0.25 g; antioxidant, 120,000 mg; and vehicle QSP., 1,000 g. 2Composition per kg of product: manganese, 75,000 mg; iron, 20000 mg; zinc, 50,000 mg; copper, 4,000 mg; cobalt, 200 mg; iodine, 1,500 mg; and vehicle QSPE, 1,000 g. 3 Butyl Hydroxy Toluene

After a 4-day adaptation period, the birds were euthanized via electrocution for ileal digesta collection. After the euthanasia process, an incision was made in the abdominal cavity to remove all the intestinal contents located 40 cm ahead of the terminal ileum just before the ileocecal junction. To ensure the quality of the collection, the birds were stimulated to consume feed prior to euthanasia to avoid excessive emptying of the digestive tract.

The ileal digesta samples were freeze-dried under vacuum at -40 °C for 72 h, and then laboratory analyses were performed to determine the amino acid profile via high-performance liquid chromatography (HPLC), as described by AOAC (1995). The dry matter and crude protein contents of the digesta were also determined according to the methodology of Silva and Queiroz (2002).

Insoluble ash and the indigestibility factor were analyzed according to the methods of Joslyn (1970). The calculation of the true ileal digestibility coefficients of amino acids was carried out via the methodology proposed by Sakomura and Rostagno (2016).

### Statistical analyses

All the collected data were subjected to analysis of variance (ANOVA) at the 5% significance level via the ExpDes.pt package in R statistical software (R Software v. 4.0.4). The normality of the residuals was first assessed via the Shapiro‒Wilk test, followed by ANOVA. The SNK (Student‒Newman‒Keuls) test was applied at the 5% significance level. For experiment 1, the statistical model adopted was:

$$\:{Y}_{ik}=\mu\:+{\tau\:}_{i}+{\epsilon\:}_{ik}$$

Where $\:{Y}_{ik}$ represents the value recorded for the response variable observed in the k-th replication of the i-th level of the tested factor, µ is the mean value recorded for the treatments, τi is the effect of the i-th level of the tested factor, and $\:{\epsilon\:}_{ik}$ is the experimental error associated with the observed $\:{Y}_{ik}$ value.

For experiment 2, the statistical model adopted was:

$$\:{Y}_{ijk}=\mu\:+{A}_{i}+{B}_{j}+{\left(AB\right)}_{ij}+{\epsilon\:}_{ijk}$$

Where $\:{A}_{i}$ is the effect of the soybean meal source, $\:{B}_{j}$ is the effect of protease inclusion, $\:{\left(AB\right)}_{ij}$ represents the interaction between these factors, and $\:{\epsilon\:}_{ijk}$ is the experimental error.

## Results

The soybean meal samples analyzed presented consistent crude protein (CP) levels, ranging from 45.87% to 47.54%, indicating a good protein content across the batches. Soluble protein and KOH solubility percentages also remained within a narrow range, with values above 79%, suggesting high protein availability for digestion. The urease activity, measured by pH variation (Δ pH), showed low values between 0.008 and 0.050, indicating that the samples were properly processed, minimizing the risk of antinutritional effects associated with urease (Table 4).

**Table 4:** Analyzed soybean meal composition, quality, and trypsin inhibitor levels

Sample	Soybean Meal CP (%)	Soluble Protein (%)	KOH Sol. (%)	TI (mg TI/g)	Urease (Δ pH)	Trypsin Inhibitors (mg IT/g)
1	46.59	37.81	81.14	2.44	0.043	8.26
2	46.80	37.69	80.55	2.44	0.029	1.23
3	46.52	37.77	81.19	2.29	0.028	1.15
4	45.98	37.53	81.64	2.16	0.017	8.96
5	47.43	37.83	79.76	2.12	0.017	0.66
6	46.11	37.03	80.31	2.70	0.050	1.63
7	45.87	37.34	81.41	2.64	0.033	3.10
8	46.82	37.89	80.92	2.76	0.008	4.38
9	47.54	38.07	80.09	2.70	0.033	1.40
10	46.65	37.68	80.79	2.48	0.029	3.42

CP (%): Crude Protein in percentage. KOH Sol. (%): Solubility of protein in potassium hydroxide solution. TI (mg TI/g): Trypsin Inhibitor activity. Urease (Δ pH): pH variation associated with urease activity. Trypsin Inhibitors (mg IT/g): Concentration of trypsin inhibitors in milligrams per gram

One of the key parameters analyzed was the concentration of trypsin inhibitors, which showed considerable variation across the samples. The value ranged from 0.66 mg IT/g (Sample 5) to 8.96 mg IT/g (Sample 4). High levels of trypsin inhibitors can negatively affect the digestibility of protein in animal feed, reducing the nutritional efficiency of the meal. Samples 1 and 4 presented the highest levels of trypsin inhibitors (8.26 mg IT/g and 8.96 mg IT/g, respectively), which may require further processing to reduce these antinutritional factors. In contrast, Samples 2, 3, 5, 6, 7, 8, 9 and 10 had lower levels of trypsin inhibitors, making them more suitable for direct use in animal nutrition.

The results of the analyses of the amino acid contents in the soybean meal are presented in Table 5. The different soybean meals evaluated in the experiment presented significant variations in apparent metabolizable energy (AME) and nitrogen-corrected apparent metabolizable energy (AMEn) values (*P* < 0.01). The soybean meals from 7, 9, 2, and 5 had the highest AME values, whereas the soybean meals from 7 to 9 had the highest AMEn values (Table 6).

**Table 5:** Analyzed digestible amino acid content of 10 samples of soybean meal from different regions as a percentage of natural matter

Amino acids	1	2	3	4	5	6	7	8	9	10
Lysine	2.99	3.19	3.15	3.34	3.34	3.34	3.37	3.37	3.24	3.33
Threonine	1.59	1.67	1.62	1.77	1.78	1.78	1.78	1.78	1.69	1.7
Methionine	0.39	0.43	0.38	0.39	0.49	0.52	0.43	0.43	0.41	0.38
Arginine	3.23	3.36	3.36	3.55	3.43	3.45	3.56	3.56	3.41	3.52
Histidine	1.1	1.11	1.1	1.25	1.1	1.14	1.23	1.23	1.18	1.16
Isoleucine	1.87	1.84	1.94	2.02	1.91	2.02	1.96	1.96	1.88	2.01
Leucine	3.52	3.58	3.64	3.81	3.85	3.86	3.74	3.74	3.63	3.75
Phenylalanine	2.16	2.16	2.26	2.34	2.24	2.34	2.31	2.31	2.24	2.32
Valine	2.01	1.95	2.08	2.16	2.02	2.16	2.13	2.13	2.00	2.17
Cystine	0.4	0.41	0.31	0.49	0.32	0.43	0.52	0.52	0.44	0.5
Alanine	2.13	2.25	2.14	2.28	2.16	2.37	2.33	2.33	2.28	2.24
Asparagine	5.84	5.98	6.04	6.35	6.33	6.40	6.31	6.31	6.00	6.22
Glutamine	7.99	8.3	8.29	8.79	8.65	8.9	8.75	8.75	8.36	8.58
Glycine	2.07	2.2	2.12	2.38	2.23	2.27	2.25	2.25	2.22	2.16
Serine	2.35	2.5	2.41	2.59	2.61	2.61	2.59	2.59	2.53	2.5
Tyrosine	1.48	1.51	1.53	1.59	1.54	1.61	1.6	1.6	1.55	1.58
Proline	2.31	2.48	2.44	2.57	2.55	2.6	2.58	2.58	2.47	2.51

**Table 6:** Mean values of apparent metabolizable energy (AME) and nitrogen balance-corrected apparent metabolizable energy (AMEn) of soybean meal with and without the inclusion of protease, based on natural matter (NM)

Soybean meal	Protease	AME, Kcal/kg de NM	AMEn, Kcal/kg of NM
1	Without	2605	2246
	With	2607	2364
2	Without	2625	2372
	With	2713	2431
3	Without	2418	2107
	With	2442	2164
4	Without	2498	2181
	With	2550	2231
5	Without	2587	2302
	With	2688	2403
6	Without	2399	2067
	With	2552	2252
7	Without	2623	2435
	With	2805	2548
8	Without	2647	2441
	With	2787	2514
9	Without	2656	2426
	With	2759	2528
10	Without	2415	2118
	With	2542	2287
Averages			
Protease	Without	2547 b	2269 b
	With	2645 a	2372 a
Soybean meal	1	2606 b	2305 c
	2	2430 d	2136 d
	3	2476 cd	2159 d
	4	2479 cd	2203 d
	5	2524 c	2206 d
	6	2714 a	2492 a
	7	2717 a	2477 a
	8	2708 a	2477 a
	9	2669 de	2401 b
	10	2638 de	2352 bc
ANOVA	Soybean meal	< 0.0001	< 0.0001
	Protease	< 0.0001	< 0.0001
P Value	Soybean meal* Protease	0.2155	0.3813
	CV (%)	3.27	3.25

Different letters in the same column indicate significant differences. SNK (*P* < 0.05). CV = coefficient of variation

The inclusion of protease resulted in significant improvements, with average increases of 97 kcal/kg and 103 kcal/kg for AME and AMEn, respectively, among the tested soybean meals (Table 6). Without the addition of the enzyme, the average AME and AMEn values were 2547 kcal/kg and 2269 kcal/kg, respectively.

With protease supplementation, these values increased to 2645 kcal/kg and 2372 kcal/kg, respectively. When the data were analyzed by location, the soybean meals from 7, 9, 2, and 5 presented the best AME and AMEn results. For example, the soybean meal from 7 presented an AME of 2717 kcal/kg and AMEn of 2477 kcal/kg, values similar to those from 7.

The addition of protease resulted in significant increases in all samples, especially for the soybean meal from 9, which presented increases of 100 kcal/kg in AME and 102 kcal/kg in AMEn. Significant variations were observed in the digestible amino acid values of the 10 soybean meals evaluated, which was attributed to differences in cultivars, planting methods, regions of origin, and industrial processing methods.

The mean ileal digestibility coefficients (IDCs) for essential amino acids, nonessential amino acids, total amino acids, lysine, methionine, methionine + cystine, threonine, and valine were 85.50%, 87.78%, 86.40%, 87.35%, 95.81%, 88.85%, 81.64%, and 85.68%, respectively (Table 7).

**Table 7:** True ileal digestibility coefficients of essential amino acids from soybean meal samples from different regions without and with protease supplementation

Soybean meal	Protease	LYS	THR	MET	ARG	HIS	With
1	Without	82.93 b	78.86	96.00	89.81	81.88 b	84,23
	With	87.31 a	81.10	96.34	89.91	83.90 a	85,95
2	Without	87.69 b	85.38 b	96.58	90.67	86.56 b	87,09
	With	93.75 a	88.53 a	97.75	90.97	89.65 a	88,37
3	Without	86.08 b	80.94 b	96.83 b	91.66 b	83.87 b	86,40 b
	With	91.84 a	87.37 a	97.89 a	93.06 a	88.24 a	89,87 a
4	Without	84.69	78.86	95.92	87.88	81.43 b	81,87
	With	85.91	80.36	96.65	88.91	84.48 a	82,37
5	Without	93.12 b	90.73	97.23	93.37	91.50	91,02
	With	95.30 a	93.20	97.52	93.70	91.98	91,66
6	Without	82.79 b	76.48 b	95.72	89.16	81.34 b	83,80 b
	With	88.52 a	80.74 a	95.82	90.30	85.87 a	87,98 a
7	Without	89.36 b	81.68 b	95.19	91.79 b	88.11 b	86,13 b
	With	96.88 a	92.63 a	95.61	95.68 a	92.11 a	91,65 a
8	Without	90.37 b	84.06 b	97.06 b	92.41 b	89.80	88,22 b
	With	94.09 a	92.32 a	98.67 a	93.92 a	90.64	92,26 a
9	Without	91.69 b	81.62 b	91.17 b	93.45	88.51	87,76
	With	93.41 a	89.03 a	96.61 a	94.45	89.60	89,43
	With	84.75 b	77.79 b	95.68 b	89.44	82.37 b	83,87 b
10	Without	88.91 a	83.56 a	97.05 a	91.37	87.49 a	87,77 a
**Protease**	Without	87.34 b	81.64 b	95.73 b	90.96 b	85.53 b	86,03 b
	With	91.59 a	86.88 a	97.00 a	92.27 a	88.40 a	88,73 a
**Soybean meal (Means values)**							
1		85.12 F	79.98 D	96.17 B	89.81 C	82.89 E	85,09 D
2		90.72 C	86.95 B	93.89 C	90.82 C	88.10 C	87,73 C
3		88.96 D	84.15 C	97.36 A	92.36 B	86.05 D	88,14 C
4		85.30 F	79.61 D	96.28 B	88.39 D	82.96 E	82.12 E
5		94.21 A	91.96 A	97.37 A	93.54 A	91.74 A	91,34 A
6		85.65 F	78.61 D	95.77 B	89.73 C	83.60 E	85.89 D
7		93.12 B	87.15 B	95.40 B	93.74 A	90.11 B	88,89 BC
8		92.22 B	88.19 B	97.83 A	93.16 DE	90.22 B	90.24 DE
9		92.55 B	85.32 BC	93.89 C	93.95 A	89.05 BC	88,59 BC
10		86.83 E	80.67 D	96.36 B	90.40 C	84.93 D	85.82 D
**CV (%)**		1.43	3.24	0.93	1.30	1.76	1,95
		***P value***					
Soybean meal		0.00001	0.00001	0.00001	0.00001	0.00001	0,00001
Protease		0.00001	0.00001	0.00001	0.00001	0.00001	0,00001
Soybean meal*Protease		0.00001	0.00042	0.00000	0.00993	0.00001	0,00002

LYS = lysine; THR = threonine; MET = methionine; ARG = arginine; HIS = histidine. Different letters indicate significant differences according to the SNK test (*P* < 0.05). CV (%): coefficient of variation

Supplementation with protease positively impacted the digestibility of amino acids, especially the key limiting amino acids such as lysine, methionine, threonine, and valine. On average, the inclusion of the enzyme resulted in a 2.94% increase in the digestibility of the evaluated amino acids. The true ileal digestibility of both essential and nonessential amino acids was increased (Tables 8 and 9). Without the enzyme, the average digestibility values were 87.34% for lysine, 81.64% for threonine, 95.73% for methionine, 90.96% for arginine, 85.53% for histidine, and 86.03% for isoleucine.

**Table 8:** True ileal digestibility coefficients of essential amino acids from soybean meal samples from different regions without and with protease supplementation

Soybean meal	Protease	LEU	PHE	VAL	AAT	AAE
1	Without	82.26	85.03	84.24	84.41	83.21
	With	82.70	86.58	85.33	85.02	84.33
2	Without	86.44	88.74	88.17	87.31	86.72 b
	With	87.10	89.51	89.23	87.48	88.80 a
3	Without	84.62	87.27 b	86.50 b	86.45 b	85.34 b
	With	85.17	90.42 a	90.18 a	89.37 a	89.00 a
4	Without	78.36	83.20	79.95	82.20 b	81.15 b
	With	79.03	83.39	80.95	84.84 a	83.64 a
5	Without	89.49	91.51	91.38	90.95	91.11
	With	90.61	91.82	92.16	91.03	91.41
6	Without	80.72	84.64 b	84.31 b	82.84 b	81.88 b
	With	81.40	87.38 a	88.03 a	86.24 a	85.38 a
7	Without	86.41	87.76 b	84.99 b	87.82 b	87.02 b
	With	87.57	92.66 a	90.46 a	91.84 a	91.87 a
8	Without	88.19	89.87 b	87.26 b	89.22	88.52 b
	With	89.48	92.23 a	92.26 a	90.73	91.43 a
9	Without	85.60	89.23 b	85.74 b	88.88	87.20 b
	With	86.31	91.46 a	88.83 a	89.39	89.44 a
10	Without	81.21	85.17 b	84.27 b	83.95 b	82.88 b
	With	82.69	88.47 a	87.49 a	87.83 a	87.07 a
**Protease**	Without	84.33	87.24 b	85.68 a	86.40 b	85.50 b
	With	85.26	89.40 a	88.50 a	88.38 a	88.24 a
**Soybean meal (Means values)**						
1		82.48 EF	85.80 C	84.78 E	84.72 EF	83.77 EF
2		86.77 CD	89.12 B	88.70 BC	87.39 D	87.76 D
3		85.90 D	88.85 B	88.34 BC	87.91 CD	87.17 D
4		87.90 F	83.29 D	80.45 F	83.52 F	82.39 F
5		90.05 A	91.67 A	91.77 A	90.99 A	91.26 A
6		86.02 EF	86.01 C	86.17 DE	84.54 EF	83.63 EF
7		87.99 BC	90.21 DE	87.72 CD	89.83 DE	89.45 BC
8		89.34 DE	91.05 A	89.76 B	89.97 DE	89.97 DE
9		86.96 CD	90.34 DE	87.29 CD	89.13 BC	88.32 CD
10		83.45 E	86.82 C	85.88 OF	85.89 E	84.97 E
**CV (%)**		2.18	1.87	2.19	1.76	1.91
		***P - Value***				
Soybean meal		0.00001	0.00001	0.00001	0.00001	0.00001
Protease		0.00001	0.00001	0.00001	0.00001	0.00001
Soybean meal*Protease		0.07763	0.01545	0.01303	0.00231	0.03624

LEU = leucine; PHE = phenylalanine; VAL = valine; AAT = total amino acids; AAE = essential amino acids; different letters differ from each other according to the SNK test (*P* < 0.05). CV (%): coefficient of variation

**Table 9:** True ileal digestibility coefficients of nonessential amino acids from soybean meal (SM) samples from different regions, with and without protease supplementation

Soybean meal	Protease	CYS	ALA	ASP	GLU	GLY	BE
1	Without	67.42 b	83.07	87.02	88.20	72.64	80.59
	With	72.37 a	84.53	87.20	88.71	73.78	80.80
2	Without	83.97	87.42	89.19	90.06	74.87 b	86.08
	With	84.19	88.54	90.77	91.44	80.33 a	87.21
3	Without	70.80 b	85.71	88.20 b	89.33 b	75.29 b	82.42 b
	With	78.59 a	86.88	91.00 a	91.73 a	79.59 a	86.66 a
4	Without	68.19 b	80.92	85.04 b	86.61 b	68.96 b	77.15 b
	With	70.05 a	81.79	87.29 a	88.48 a	78.94 a	81.55 a
5	Without	88.91 b	91.38	92.40	92.48	83.02	89.39
	With	94.03 a	91.49	93.25	92.81	84.68	90.08
6	Without	72.24 b	82.39	84.94 b	86.88 b	69.31 b	77.13 b
	With	78.74 a	82.49	89.91 a	90.28 a	74.28 a	80.87 a
7	Without	89.90	87.39	89.62 b	90.85 b	82.24	85.28 b
	With	91.69	88.64	93.57 a	94.52 a	84.85	89.05 a
8	Without	87.21 b	88.71	90.54	92.29	82.24	87.02 b
	With	94.60 a	89.53	91.79	93.34	82.74	89.64 a
9	Without	90.95 b	85.76	91.29	93.05	76.88 b	84.58 b
	With	95.15 a	86.20	92.08	93.08	78.02 a	87.34 a
10	Without	67.98 b	83.11	86.66 b	88.44 b	72.24 b	78.92 b
	With	77.78 a	84.26	90.62 a	90.78 a	79.56 a	84.43 a
Protease	Without	78.75 b	85.59	88.49 b	89.81 b	75.76 b	82.85 b
	With	83.71 a	86.44	90.75 a	91.52 a	79.68 a	85.76 a
**Soybean meal (Means values)**							
1		68.74 F	83.80 DE	87.11 E	88.45 CD	73.21 D	80.69 OF
2		87.92 C	87.99 ABCD	89.98 C	90.75 B	77.60 B	86.64 BC
3		72.95 E	87.29 ABCD	89.60 CD	90.53 B	77.44 B	84.54 C
4		72.55 E	82.86 E	86.16 E	87.55 D	73.95 CD	79.35 E
5		91.47 B	91.44 A	92.82 A	92.65 A	83.85 A	89.73 A
6		70.54 EF	84.34 OF	87.43 E	88.63 CD	71.79 D	78.99 E
7		90.79 B	89.02 ABC	91.59 DE	92.68 A	83.54 A	87.17 B
8		93.82 A	89.62 DE	91.16 B	92.81 A	82.49 A	88.33 DE
9		87.92 C	86.48 BCDE	91.68 B	93.06 A	77.44 B	85.95 BC
10		65.73 G	85.19 CDE	88.64 D	89.61 BC	75.90 BC	81.68 D
**CV (%)**		3.37	4.30	1.54	1.40	3.40	2.66
		***P value***					
Soybean meal		0.00001	0.00001	0.00001	0.00001	0.00001	0.00001
Protease		0.00001	0.00001	0.00001	0.00001	0.00001	0.00001
Soybean meal*Protease		0.00001	0.99999	0.00001	0.00458	0.00001	0.01963

CYS = cysteine; ALA = alanine; ASP = aspartic acid; GLU = glutamic acid; GLY = glycine. CV (%): coefficient of variation. Different letters in the same column indicate significant differences. SNK (*P* < 0.05)

With enzyme supplementation, these values increased to 91.59%, 86.88%, 97.00%, 92.27%, 88.40%, and 88.73%, respectively. The average increases in digestibility with protease supplementation were 4.25% for lysine, 1.22% for threonine, 3.09% for methionine, 5.24% for valine, 2.81% for methionine + cystine, 2.73% for essential amino acids, 2.12% for nonessential amino acids, and 2.03% for total amino acids. For nonessential amino acids such as leucine, phenylalanine, valine, cysteine, alanine, aspartic acid, glutamic acid, glycine, and serine, the average digestibility values also increased with increasing addition of the enzyme (Table 9).

A significant effect (*P* < 0.05) of treatment on the true ileal digestibility coefficient (TIDC) of the nonessential amino acids methionine + cystine, phenylalanine + tyrosine, and glycine + serine was observed among the soybean meals from different regions, with and without enzyme supplementation (Table 10). The addition of protease resulted in average increases in digestibility for the main amino acids, with increases ranging from 0.08% to 10.95% (Table 11). Overall, the enzyme improved the digestibility of all the amino acids evaluated.

**Table 10:** True ileal digestibility coefficients of nonessential amino acids. methionine + cystine. phenylalanine + tyrosine and glycine + serine from soybean meal samples from different regions. With and Without protease supplementation

Soybean meal	Protease	TYR	FOR	AANE	MET + CYS	PHE + TYR	GLY + SER
1	Without	84.15	81.41	85.99	85.05 b	84.69	77.01
	With	85.57	83.14	86.69	88.24 a	86.19	77.44
2	Without	88.57 b	82.83	88.50	90.19	88.67 b	80.97 b
	With	90.44 a	83.65	89.85	90.31	89.88 a	84.07 a
3	Without	86.74 b	82.11 b	87.20 b	84.82 b	87.06 b	79.17 b
	With	89.86 a	87.38 a	90.43 a	92.11 a	90.20 a	83.40 a
4	Without	82.94	79.54 b	84.33 b	87.53 b	83.10	73.38 b
	With	84.61	83.16 a	86.57 a	89.96 a	83.87	80.35 a
5	Without	92.36	87.19	91.78	92.14 b	91.96	86.01
	With	92.66	87.19	92.17	95.44 a	92.04	88.10
6	Without	83.66 b	77.09 b	84.06 b	81.75 b	84.26 b	73.55 b
	With	86.64 a	82.56 a	88.44 a	90.48 a	87.09 a	77.85 a
7	Without	87.46 b	85.80	89.30 b	93.54	87.64 b	83.89 b
	With	92.47 a	88.19	92.90 a	93.97	92.59 a	87.14 a
8	Without	89.68 b	85.62	90.59	94.70 b	89.80 b	85.11
	With	92.65 a	86.75	91.82	96.60 a	92.40 a	86.34
9	Without	88.84 b	82.75	90.63	89.82 b	89.07 b	81.65
	With	91.14 a	85.04	90.66	92.23 a	91.33 a	82.54
10	Without	84.29 b	78.95 b	85.38 b	81.68 b	84.82 b	75.87 b
	With	88.49 a	86.06 a	89.44 a	90.91 a	88.48 a	82.19 a
**Protease**	Without	86.86 b	82.32 b	87.77 b	88.12 b	84.57 b	79.66 b
	With	89.45 a	85.31 a	89.90 a	92.02 a	89.41 a	82.94 a
**Soybean meal (Means values)**							
1		84.86 OF	82.28 CD	86.34 CD	86.64 E	85.44 D	77.22 CD
2		89.51 BC	83.24 CD	89.18 B	90.25 C	89.27 C	82.52 B
3		88.30 C	84.74 BC	88.81 B	88.46 D	88.63 C	81.28 B
4		83.77 E	81.35 DE	85.44 D	88.74 D	83.48 E	76.86 CD
5		92.51 A	87.93 A	91.98 A	93.79 B	91.99 A	87.06 A
6		85.15 OF	79.82 E	86.25 CD	86.11 E	85.67 D	75.70 D
7		89.96 BC	86.99 A	91.10 A	93.75 B	90.11 BC	85.51 A
8		91.17 B	86.19 A	91.20 A	95.75 A	91.10 DE	85.73 A
9		89.99 BC	83.90 C	90.65 A	91.02 C	90.20 BC	82.09 B
10		86.39 D	82.50 CD	87.41 C	86.29 E	86.65 D	79.03 C
**CV (%)**		1.85	2.62	1.61	1.43	1.84	2.84
		***P value***					
Soybean meal		0.00001	0.00001	0.00001	0.00001	0.00001	0.00001
Protease		0.00001	0.00001	0.00001	0.00001	0.00001	0.00001
Soybean meal* Protease		0.01259	0.00002	0.00040	0.00001	0.01637	0.00005

Tyr = tyrosine; Pro = proline; Nane = nonessential amino acids; Met + Cys = methionine + cystine; Phe + Tyr = phenylalanine + tyrosine; GLY + SER = glycine + serine. Different letters differ from each other according to the SNK test (*P* < 0.05). CV (%): coefficient of variation

**Table 11:** Average percentage Increase In the digestibility of the main amino acids promoted by the Inclusion of the protease which is based on natural matter (NM). In soybean meals from different regions

Soybean meal	Lys	Thr	Met	Val	Met + Cys	EAA	NEA	TAA
1	4.38	2.24	0.34	1.09	3.19	1.12	0.7	0.61
2	6.06	3.15	0.83	1.06	0.12	2.08	1.35	0.17
3	5.76	6.43	1.06	3.68	7.29	3.66	3.23	2.92
4	1.22	1.50	0.73	1.00	2.43	2.49	2.24	2.64
5	2.18	2.47	0.29	0.78	3.3	0.3	0.39	0.08
6	5.73	4.26	0.10	3.72	8.73	3.5	4.38	3.4
7	7.52	10.95	0.42	5.47	0.43	4.85	3.6	4.02
8	3.72	8.26	1.61	5.00	2.1	2.91	1.23	1.51
9	1.72	7.41	5.44	3.09	2.41	2.24	0.03	1.05
10	4.16	5.77	1.37	3.22	0.95	4.19	4.06	3.88
Overall Average (%)	4.25	1.22	3.09	5.24	2.81	2.73	2.12	2.03

Lys = lysine. Thr = threonine. Met = methionine. Val = valine. Met + Cys = methionine + cystine. EAA = essential amino acids. NEA = nonessential amino acids. TAA = total amino acids

## Discussion

The results of this study demonstrate that supplementation with the protease in soybean meals significantly improves both apparent metabolizable energy (AME) and nitrogen-corrected apparent metabolizable energy (AMEn), in addition to promoting increases in the digestibility of essential and nonessential amino acids. On average, the soybean meals without enzyme addition had AME values of 2547 kcal/kg and AMEn values of 2269 kcal/kg, whereas with enzyme supplementation, these values increased to 2645 kcal/kg and 2372 kcal/kg, respectively. These findings are consistent with the literature that describes the benefits of enzyme supplementation in animal feed (Adeola and Cowieson 2011; Ravindran et al. 2014).

The effectiveness of supplementation with proteolytic enzymes, such as proteases, has been widely documented, particularly in terms of improving the metabolizable energy of feed ingredients. According to Ravindran et al. (1999), enzymes increase nutrient availability by breaking chemical bonds that hinder the digestibility of food components.

In this study, the average increases of 97 kcal/kg for AME and 103 kcal/kg for AMEn support findings from other studies, which reported increases in the range of 50–150 kcal/kg with enzyme supplementation (Cowieson and Adeola 2005; Angel et al. 2011). These results are also in line with the observations of Walk et al. (2012a), who highlighted the positive effects of proteases on nutrient digestibility.

In addition to improvements in metabolizable energy, the true ileal digestibility of essential and nonessential amino acids was significantly enhanced with enzyme inclusion. In particular, the digestibility of limiting amino acids such as lysine, threonine, methionine, and valine substantially increased, which aligns with the findings of Walk et al. (2012b) and other studies reporting improvements ranging from 2% to 6% in the digestibility of essential amino acids with enzyme supplementation (Nunes et al. 2012). For example, in this study, the digestibility of lysine increased from 87.34% to 91.59%, values comparable to those reported in the literature. Jabbar et al. (2021), studying diets with different protein levels (17, 19, and 21%) supplemented with proteases in initial diets for broiler chickens, also found an improvement in AME and digestibility of crude protein in the diet, regardless of the protein level studied in the diet.

Previous studies, such as those by Ghazi et al. (2002) and Xavier et al. (2024), also demonstrated improvements in the digestibility of amino acids such as lysine and methionine with protease use in poultry diets. These improvements reinforce the potential of enzyme supplementation to optimize the use of soybean meals in animal nutrition.

The variation in digestibility and metabolizable energy values among the different soybean meals analyzed can be attributed to factors such as differences in cultivars, agricultural conditions, and industrial processing methods. Silva et al. (2022) and Liu et al. (2024) described how these factors can significantly influence the quality of soybean meal. In this study, soybean meals from 7 to 9 presented the highest AME and AMEn values, suggesting that regional characteristics and processing practices play important roles in determining the nutritional quality of soybean meal (Kumar et al. 2010; Ravindran et al. 2014). This point is also supported by Cowieson et al. (2006), who mention regional variations as critical factors in ingredient quality and enzyme additive efficacy.

Odetallah et al. (2002) reported substantial increases in the digestibility of essential amino acids with enzyme supplementation, which corroborates the findings of this study. Vieira et al. (2022) reported the positive effects of including the protease were particularly pronounced for the limiting amino acids. Significant improvements in the digestibility of methionine, lysine, threonine, and valine were observed, confirming results reported in studies such as those by Olukosi et al. (2010) and Cowieson and Roos (2016).

Increases in the apparent ileal digestibility coefficients of CP, cysteine, histidine, isoleucine, leucine, phenylalanine, tyrosine, valine, glutamic acid, glycine, proline, and serine were also observed by Qiu et al. (2023) when supplementing broiler diets with 250 mg/kg of protease at 42 days of age.

These results are consistent with the scientific literature that emphasizes the benefits of enzyme supplementation in animal feed (França et al. 2023; Jacob et al. 2021). Supplementation with the protease significantly increased energy metabolism and amino acid digestibility in soybean meals from different regions. According to Liu et al. (2024), the proteases have been shown to be effective in increasing the nutritional value of soybean meal, especially in batches with intrinsically lower digestibility, contributing to economic gains in animal production.

These improvements are especially relevant for animal nutrition, as they optimize the utilization of nutrients in soybean meals, increasing their feed efficiency and nutritional value (Adeola and Cowieson 2011; Ravindran et al. 2014; Xavier et al. 2024). Furthermore, protease supplementation in wheat and soybean meal-based diets was observed to reduce taurine concentrations in jejunal digesta (1097 vs. 870 mg/kg) in broilers, indicating reduced bile acid secretion without reducing the rate of lipid digestion and absorption. This results in lower energy and protein expenditure for bile production and secretion, resulting in an indirect gain (Cowieson et al. 2017).

Supplementation with the protease has proven to be an effective strategy for improving the metabolizable energy and amino acid digestibility of the soybean meals evaluated, with a particular emphasis on the soybean meals from 7 to 9, which presented the best nutritional performance. Therefore, supplementation with the protease represents a promising alternative for improving feed efficiency and sustainability in animal production, especially in diets based on soybean meals from different origins. A enzymatic supplementation has been recognized nutritionally, environmentally, and economically. For optimal growth, young birds require more digestible protein. Due to the complex nature of soy protein, its rapid passage through the digestive system, and the deficiency of innate enzymes, protein digestibility can be significantly affected. Therefore, the addition of protease enzymes is essential in broiler diets during the initial phase.

## Conclusions

Soybean meals from different regions exhibit significant variations in terms of metabolizable energy and digestible amino acids, and the addition of protease improves both the digestibility and true ileal digestibility coefficients, resulting in increased AME and AMEn values.

## Data Availability

The datasets generated and/or analyzed during the current study are not publicly available because only the processed data (means and results of the statistical analyses) are presented in the manuscript. However, the raw data that support these findings are available from the corresponding author upon reasonable request.
